# Solid-pseudopapillary neoplasm of the pancreas in a patient with familial adenomatous polyposis: a case report

**DOI:** 10.1186/s40792-021-01121-x

**Published:** 2021-01-28

**Authors:** Daishi Naoi, Koji Koinuma, Hideki Sasanuma, Yasunaru Sakuma, Hisanaga Horie, Alan Kawarai Lefor, Kokichi Sugano, Mineko Ushiama, Teruhiko Yoshida, Naohiro Sata

**Affiliations:** 1 Division of Gastroenterological, General and Transplant Surgery, Department of Surgery, Jichi Medical University School of Medicine, Yakushiji 3311-1, Shimotsuke, Tochigi 329-0498 Japan; 2 Genome Center, Genetic Counseling Clinic, Tochigi Cancer Center Research Institute, Tochigi, Japan; 3 Department of Genetic Medicine and Services, National Cancer Center Hospital, Tokyo, Japan

**Keywords:** Solid-pseudopapillary neoplasm, Familial adenomatous polyposis, Laparoscopic surgery, Case report

## Abstract

**Background:**

Familial adenomatous polyposis (FAP) is characterized by the presence of hundreds to thousands of colonic polyps, and extracolonic manifestations are likely to occur. Pancreatic tumors are rare extracolonic manifestations in patients with FAP, among which solid-pseudopapillary neoplasm (SPN) are extremely rare. We report here a patient with an SPN of the pancreas found during the follow-up of FAP.

**Case presentation:**

A 20-year-old woman was diagnosed with FAP 3 years previously by colonoscopy which revealed less than 100 colonic polyps within the entire colon. She complained of left upper abdominal pain and a 10-cm solid and cystic pancreatic tumor was found by computed tomography scan. Solid and cystic components within the tumor were seen on abdominal magnetic resonance imaging. Simultaneous laparoscopic resection of the distal pancreas and subtotal colectomy was performed. Histopathological findings confirmed the pancreatic tumor as an SPN without malignancy. Abnormal staining of beta-catenin was observed by immunohistochemical study. Multiple polyps in the colorectum were not malignant. Molecular biological analysis from peripheral blood samples revealed a decrease in the copy number of the promoter 1A and 1B region of the *APC* gene, which resulted in decreased expression of the *APC* gene.

**Conclusions:**

A rare association of SPN with FAP is reported. The genetic background with relation to beta-catenin abnormalities is interesting to consider tumor development. So far, there are few reports of SPN in a patient with FAP. Both lesions were treated simultaneously by laparoscopic resection.

## Background

Familial adenomatous polyposis (FAP) is an autosomal dominant disease characterized by the presence of hundreds to thousands of adenomatous polyps in the colorectum, and colon cancer is considered inevitable over a lifetime. Extracolonic manifestations are likely to occur in patients with FAP [1, 2]. Pancreatic tumors are rare extracolonic manifestations in patients with FAP [3, 4], and there are few reports of solid-pseudopapillary neoplasms (SPN) in patients with FAP [5–7].

We report a patient with FAP who was diagnosed with a SPN during the follow-up, and both diseases were treated simultaneously by laparoscopic resection.

## Case presentation

**Fig. 1:**
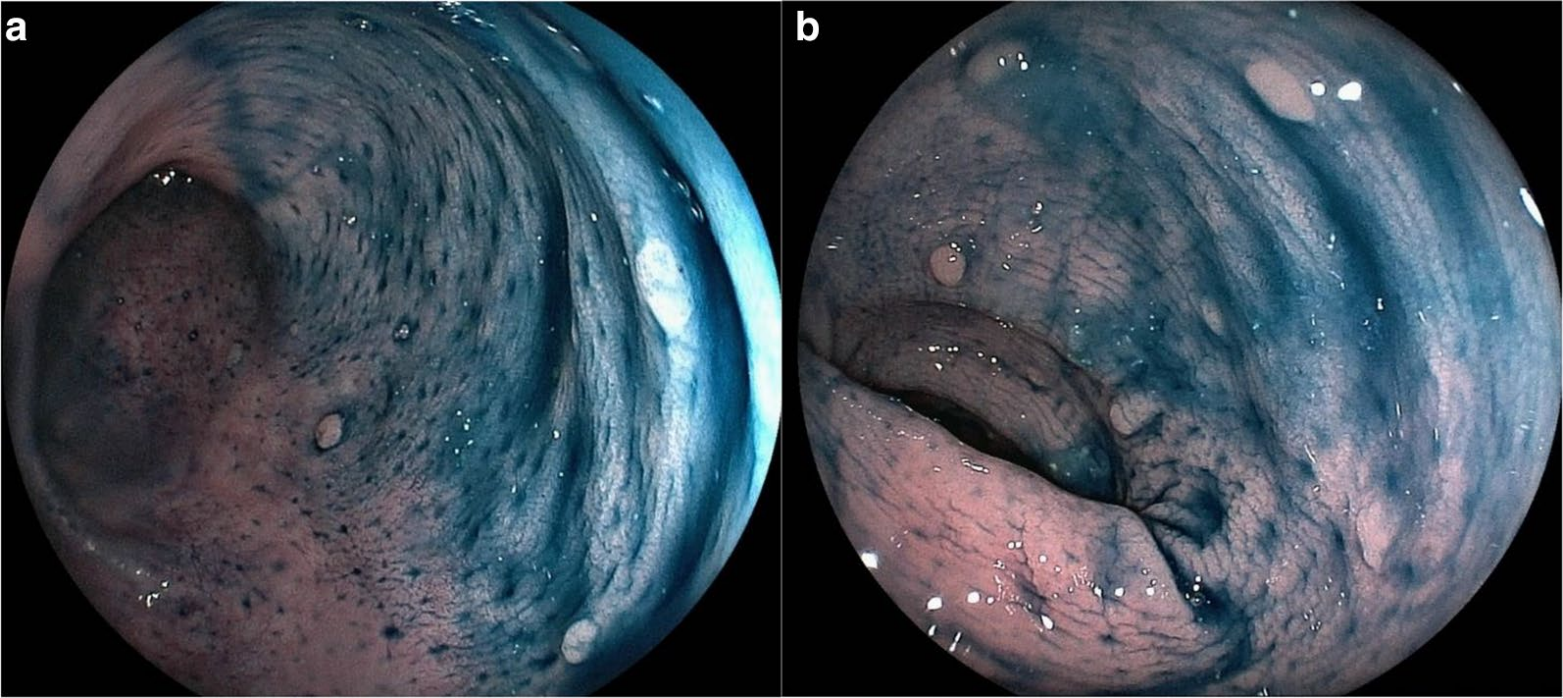
Colonoscopy showed less than 100 benign polyps involving the entire colon (**a**, **b**)

**Fig. 2:**
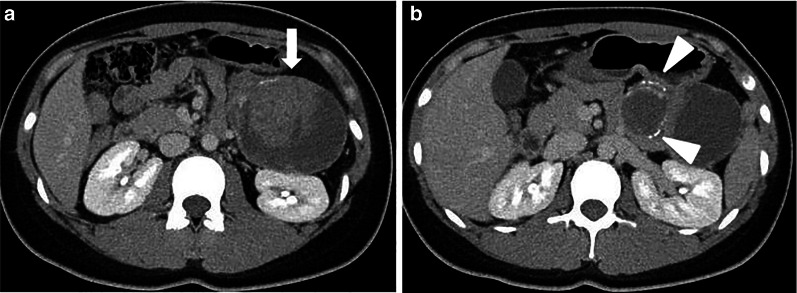
Abdominal computed tomography scan images. A 10-cm clearly demarcated tumor (arrow) with solid and cystic components are seen in the pancreatic tail (**a**), and the capsule had calcifications (arrowhead) (**b**)

**Fig. 3:**
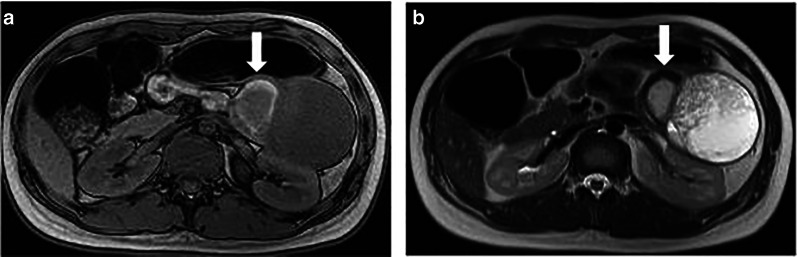
Magnetic resonance imaging. The tumor had bleeding (arrow) shown by high intensity on T1-weighted images (**a**), and a low intensity on T2-weighted images (**b**)

The patient is a 20-year-old woman diagnosed with FAP 3 years previously by colonoscopy which revealed less than 100 benign polyps involving the entire colon (Fig. 1a, b). Her father, older brother and older sister were all diagnosed with FAP and treated by surgical resection. Her older brother and older sister had desmoid tumors. She presented with left upper abdominal pain and enhanced computed tomography scan showed a 10-cm pancreatic tumor with clear margins defined by a calcified capsule. Solid and cystic components were inside the lesion (Fig. 2a, b). On magnetic resonance imaging, part of the cystic component had high intensity on T1- (Fig. 3a) and low intensity on T2-weighted images (Fig. 3b), suggesting intra-tumor bleeding. No signs of malignancy were observed. The pancreatic tumor was diagnosed as an SPN. Given that she was already 20 years old from a FAP family, prophylactic colectomy was indicated. Simultaneous laparoscopic distal pancreatectomy and subtotal colectomy with an ileo-rectal anastomosis were performed. The post-operative course was uneventful. She remains free of SPN recurrence 5 years postoperatively, although an abdominal desmoid tumor is followed up.

**Fig. 4:**
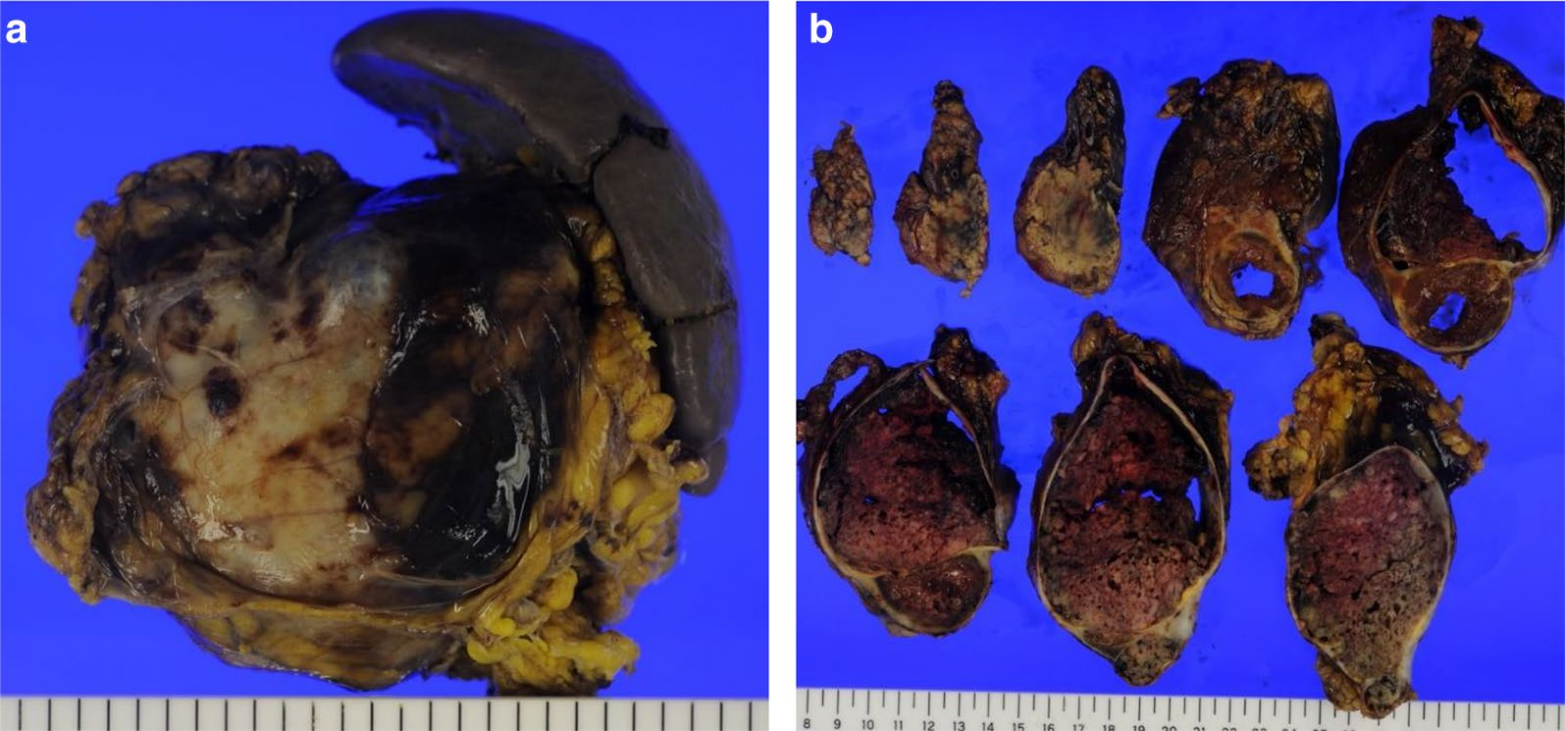
Resected specimen. Gray solid component with bleeding and a multi-locular cystic component with necrosis were inside a thick capsule (**a**, **b**)

**Fig. 5:**
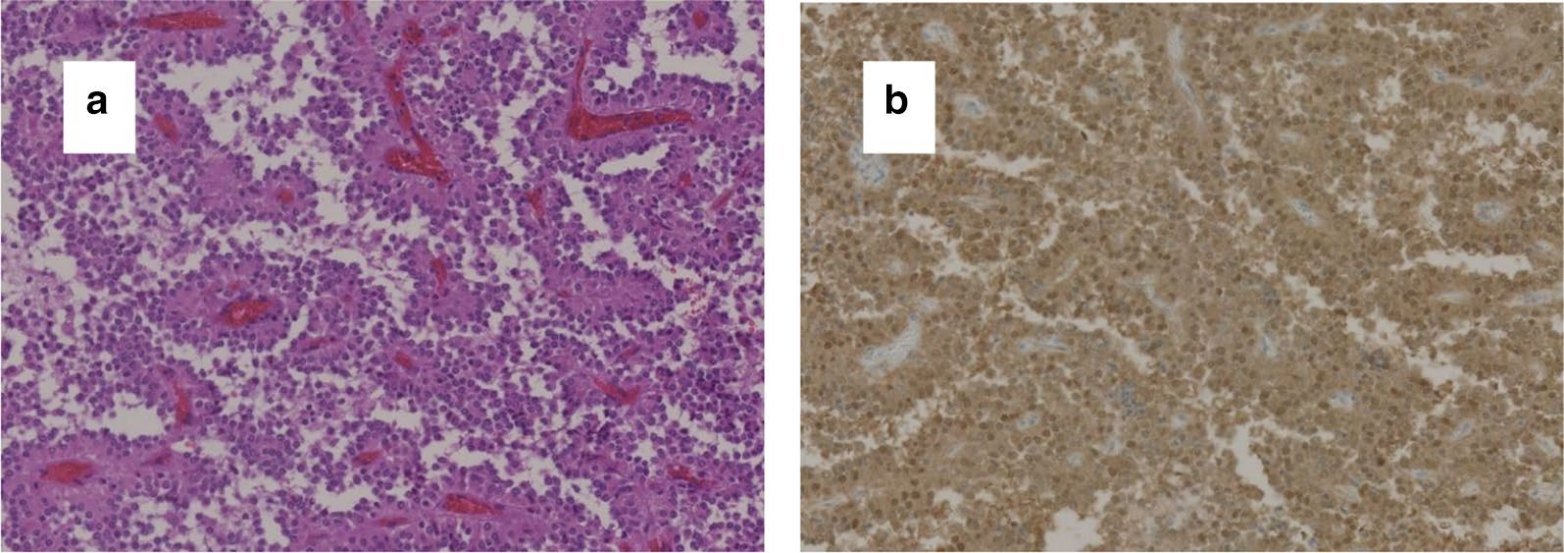
Pathological findings of the pancreatic tumor (a; hematoxylin & eosin × 400). Immunohistochemical examination shows both nucleus and cytoplasm positive for β-catenin (b; × 400)

Macroscopically, the SPN had a gray solid component with bleeding and a multi-locular cystic component with necrosis (Fig. 4a, b). Histopathological findings of the SPN revealed loose tumor cell connectivity with cell dissociation and pseudopapillary arrangements (Fig. 5a). There was no evidence of malignancy. Immunohistochemical staining showed that both the nucleus and cytoplasm in the tumor cells stained with beta-catenin (Fig. 5b) but were negative for chromogranin A and synaptophysin. The polyps in the colorectum had no evidence of malignancy.

**Fig. 6:**
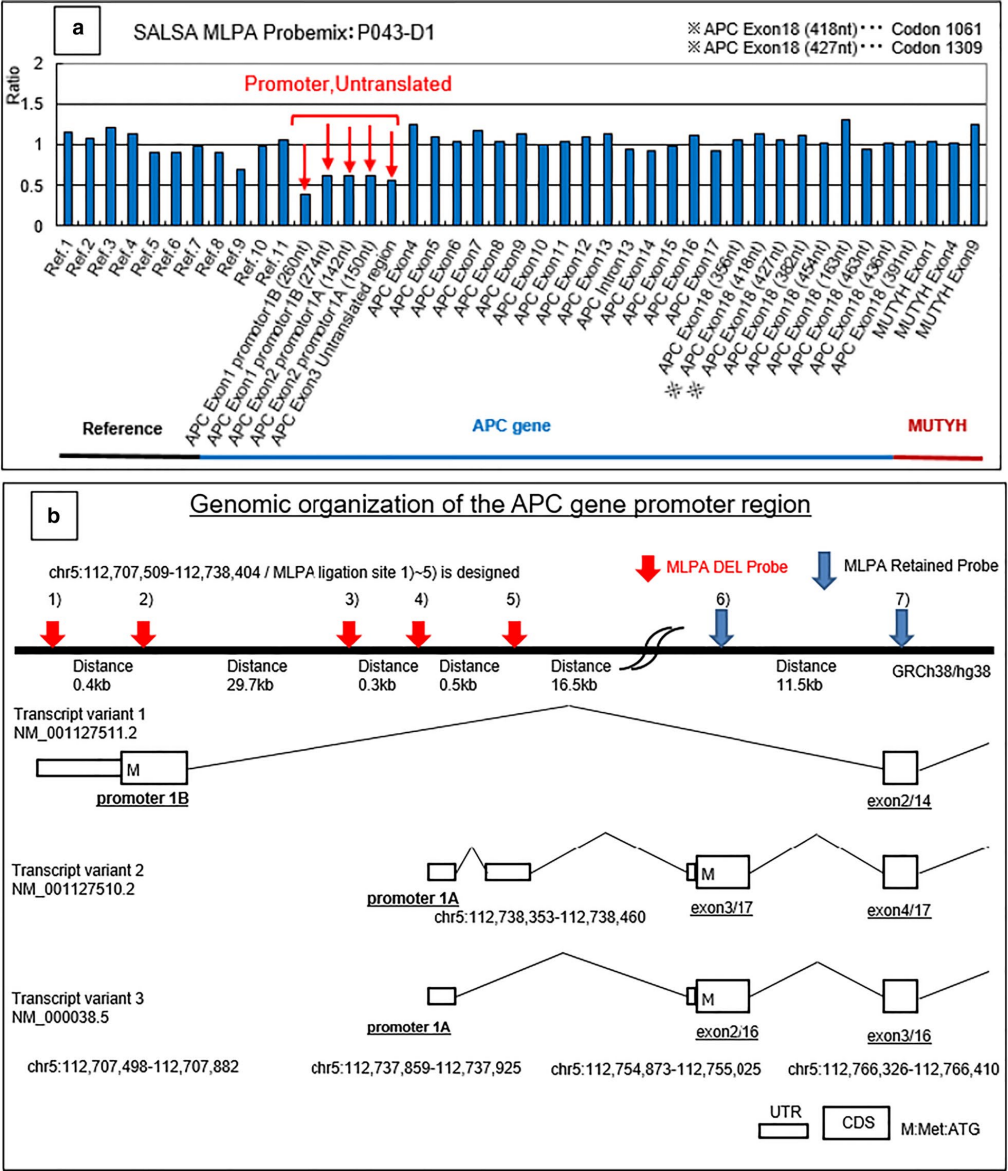
Analysis by the multiplex ligation-dependent probe amplification technique. A decrease in the copy number of the promoter 1A and 1B region of the *APC* gene was revealed (**a**). Genomic organization of the promoter region of the APC genes and arrows indicate the positions of probes hybridizing the APC gene (**b**)

Protein truncation tests from an analysis of peripheral blood samples revealed no mutations in the protein coding region of the *APC* (adenomatous polyposis coli) gene. Analysis by the multiplex ligation-dependent probe amplification (MLPA) technique that can quantitatively analyze exonic deletions and duplication revealed a decrease in the copy number of the promoter 1A and 1B region of the *APC* gene (Fig. 6a, b), which resulted in decreased expression of the *APC* gene in this patient [8]. Genetic analysis of the resected specimen was not performed.

## Discussion

FAP is caused by mutations of the *APC* gene. Loss of function mutation in the *APC* gene is associated with an accumulation of intracellular beta-catenin protein, which leads to loss of control of normal beta-catenin signaling, which promotes colorectal adenoma formation. If prophylactic colectomy is not performed, colon cancer is considered inevitable over a lifetime.

Extracolonic manifestations are likely to occur in patients with FAP [1, 2], including benign (osteomas, epidermoid cysts, desmoid tumor, congenital hypertrophy of the retinal pigment epithelium, fundic gland polyposis) and malignant (adenocarcinoma of the duodenum, thyroid, pancreas, biliary tract, stomach, and tumor of the liver or central nervous system) lesions [1–3, 9, 10]. Pancreatic tumors are rare extracolonic manifestations in patients with FAP, most of which were reported to be ductal adenocarcinoma of the pancreas. Although, the life-time risk of pancreatic carcinoma is estimated at about 2% in patients with FAP, the risk has been estimated to be more than four times that of the general population [3, 4]. However, there are few reports of SPN in patients with FAP [5–7]. SPN is a rare tumor with low malignant potential, representing about 0.17 to 2.7% of pancreatic tumors [11]. Most are seen in young females and are in the body or tail of the pancreas. Surgical resection is recommended for treatment, as 95% or more can be cured by complete resection [12–14]. Genetically, *beta-catenin* mutations frequently occur in patients with SPN and has been thought to play a critical role in the tumorigenesis of SPN. An abnormal nuclear accumulation of beta-catenin protein is observed in almost 100% of SPN lesions [15, 16]. It is interesting that beta-catenin abnormalities are observed in both SPN and FAP, although their association in tumorigenesis is still unclear.

In previously reported patients with FAP who develop SPN, the SPN were diagnosed during follow-up of colon cancer in two patients [5, 6]. The other was a 14-year-old girl who had a history of SPN resection and was subsequently diagnosed with FAP [7]. In the present patient, SPN was diagnosed during the follow-up of FAP. Our institution has sufficient experience performing laparoscopic colectomy and laparoscopic distal pancreatectomy, so we decided to perform laparoscopic resection of both lesions. Precise preoperative diagnoses made it possible to perform a minimally invasive surgery.

This patient had been followed as attenuated FAP (AFAP) due to the small number of colonic polyps seen on colonoscopy. AFAP is characterized by the presence of less than 100 colonic polyps, more proximally located, and a later age at diagnosis of colon cancer compared to classic FAP. AFAP is estimated to represent about 8–15% of all FAP, and its clinical phenotype is milder with a lower incidence of extracolonic manifestations [1, 17]. Siblings of this patient developed desmoid tumors, which also developed in this patient. MLPA analysis elucidated a decrease in the copy number of the promoter 1A and 1B region of the *APC* gene, which was different from previous reports in which the mutations in the *APC* gene associated with AFAP have mainly been presented in the 5′ upstream exons, in exon 9 and in the distal 3′ end [15, 18]. Accordingly, this patient is likely to be classical FAP despite the small number of colonic polyps. It is difficult to assess whether a patient has AFAP at a younger age, careful follow-up including the occurrence of extracolonic manifestations is required.

## Conclusion

We report a patient with SPN and FAP. The genetic background of the lesions with relation to beta-catenin abnormalities is interesting to consider tumor development and coexistence. There have been few previous case reports of SPN in a patient with FAP, and both diseases were treated simultaneously by laparoscopy.

## Data Availability

Not applicable.

## Abbreviations

FAP: Familial adenomatous polyposis
SPN: Solid-pseudopapillary neoplasm
*APC*: Adenomatous polyposis coli
MLPA: Multiplex ligation-dependent probe amplification
AFAP: Attenuated familial adenomatous polyposis
